# Meridianins Rescue Cognitive Deficits, Spine Density and Neuroinflammation in the 5xFAD Model of Alzheimer’s Disease

**DOI:** 10.3389/fphar.2022.791666

**Published:** 2022-02-24

**Authors:** Ened Rodríguez-Urgellés, Anna Sancho-Balsells, Wanqi Chen, Laura López-Molina, Ivan Ballasch, Ignacio del Castillo, Conxita Avila, Jordi Alberch, Albert Giralt

**Affiliations:** ^1^ Departament de Biomedicina, Facultat de Medicina, Institut de Neurociències, Universitat de Barcelona, Barcelona, Spain; ^2^ Institut d’Investigacions Biomèdiques August Pi i Sunyer (IDIBAPS), Barcelona, Spain; ^3^ Centro de Investigación Biomédica en Red Sobre Enfermedades Neurodegenerativas (CIBERNED), Barcelona, Spain; ^4^ Department of Evolutionary Biology, Ecology and Environmental Sciences, Faculty of Biology and Biodiversity Research Institute (IRBio), Universitat de Barcelona, Catalonia, Spain; ^5^ Production and Validation Center of Advanced Therapies (Creatio), Faculty of Medicine and Health Science, University of Barcelona, Barcelona, Spain

**Keywords:** microglia, GSK3β, learning, memory, astrocytes

## Abstract

Glycogen synthase kinase 3β (GSK3β) is a core protein, with a relevant role in many neurodegenerative disorders including Alzheimer’s disease. The enzyme has been largely studied as a potential therapeutic target for several neurological diseases. Unfortunately, preclinical and clinical studies with several GSK3β inhibitors have failed due to many reasons such as excessive toxicity or lack of effects in human subjects. We previously reported that meridianins are potent GSK3β inhibitors without altering neuronal viability. In the present work, we examine whether meridianins are capable to inhibit neural GSK3β *in vivo* and if such inhibition induces improvements in the 5xFAD mouse model of Alzheimer’s Disease. Direct administration of meridianins in the third ventricle of 5xFAD mice induced robust improvements of recognition memory and cognitive flexibility as well as a rescue of the synaptic loss and an amelioration of neuroinflammatory processes. In summary, our study points out meridianins as a potential compound to treat neurodegenerative disorders associated with an hyperactivation of GSK3β such as Alzheimer’s disease.

## 1 Introduction

Alzheimer’s disease (AD), the most common type of dementia affecting millions of people worldwide, is characterized by progressive cognitive impairment, typically beginning with memory deterioration and followed by executive dysfunction and language, visual and practical problems along with emotional and psychiatric symptoms (Bondi et al., 2017; Long and Holtzman, 2019). AD pathology starts in structures such as the hippocampus and the entorhinal cortex (DeTure and Dickson, 2019; Long and Holtzman, 2019), being the extracellular amyloid-β (Aβ) plaques and intracellular tangles of abnormally hyperphosphorylated Tau the most representative AD hallmarks (DeTure and Dickson, 2019). Aβ accumulation over the course of the disease impair synaptic plasticity, activate an inflammatory response, and compromise neuronal survival (Mucke and Selkoe, 2012; Tracy and Gan, 2018). Neuroinflammation has an active role in AD pathogenesis (Hauss-Wegrzyniak et al., 1998; McGeer et al., 2000; Simón-Sánchez et al., 2009) being an overreactivity of astrocytes and microglia a principal neuropathological event of the disease and potentially affecting neuronal connectivity too (Heneka et al., 2015; Leng and Edison, 2021). The study of potential treatments for this neurodegenerative disease during the last two decades has been unfruitful. In the field of AD, there are currently four approved compounds whereas more than 100 potential compounds have been abandoned at different phases of clinical trials (Mehta et al., 2017).

One of the major molecular mechanisms underlying AD that has been object of intense investigation is the classical glycogen synthase kinase 3 β (GSK3β) pathway (Hooper et al., 2008). GSK3β is a ubiquitous serine (Ser)/threonine (Thr) protein kinase involved in the transfer of a phosphate group from adenosine triphosphate (ATP) to Ser and threonine acid residues of target substrates. GSK3β is constitutively active; its substrates usually need to be prephosphorylated by another kinase, and it is inhibited, rather than activated, in response to stimulation (Ding et al., 2000; Hooper et al., 2008; Palsgaard et al., 2012). The predominant hypothesis in AD suggests that GSK3β is affected by amyloid peptides (Hooper et al., 2008). Changes in the kinase activity of GSK3β alter synaptic signals essential for learning and memory processes (Kremer, 2011). GSK3β activity can be regulated by serine 9 phosphorylation (Hooper et al., 2008). In AD, GSK3β is commonly regulated by inhibitory phosphorylation on Ser9, located at the N-terminal tail. The dysregulation of this process results in a GSK3β permanent abnormal activation that in turn induces memory impairment, increased production of Aβ, and inflammatory responses (Hooper et al., 2008). Despite the relevance of GSK3β as a potential therapeutic target, several inhibitors of the kinase have failed at several steps of preclinical and clinical studies being the lack of effects or excessive toxicity among the main causes (Bhat et al., 2018; del Ser et al., 2012; Hampel et al., 2009; Kreisl et al., 2009; Macdonald et al., 2008; Nwankwo et al., 2013; Tolosa et al., 2014; Zamek-Gliszczynski et al., 2014).

Meridianins are a family of indole alkaloids isolated from marine benthic organisms from Antarctica (Núñez-Pons et al., 2010; Núñez-Pons et al., 2012). These ascidian’s natural products consist of an indole framework linked to an aminopyrimidine ring. These compounds are isolated from specimens of the tunicate genus *Aplidium* Savigny, 1816 (Núñez-Pons et al., 2010; Núñez-Pons et al., 2012). In a previous report, we showed, by using docking calculations and molecular dynamic simulations, the ability of meridianins to act as ATP-competitive or non-ATP-competitive inhibitors of GSK3β (Llorach-Pares et al., 2020). In the same study, we also demonstrated the capacity of meridianins to inhibit GSK3β *in vitro* without altering the neuronal survival. In the present study, we hypothesized that meridianins have the potential to inhibit GSK3β *in vivo* and that such inhibition may improve cognitive decline in AD. To demonstrate our hypothesis, we tested whether meridianins are capable to correct learning and memory deficits, neuroinflammatory processes, and synapse loss in the 5xFAD mouse model of AD.

## 2 Material and Methods

### 2.1 Animals

For this study, we used the transgenic mouse line 5xFAD (MMRRC catalog #034840-JAX). Nine-month-old 5xFAD mice overexpressing the 695-amino acid isoform of the human amyloid precursor protein (APP695) carrying the Swedish, London, and Florida mutations under the control of the murine Thy-1 promoter were used. In addition, they express human presenilin-1 (PSEN-1) carrying the M146L/L286V mutation, also under the control of the murine Thy-1 promoter (Oakley et al., 2006). Also, for experiments of inhibition of hippocampal activity, we used 9-month-old C57BL6/J male mice (catalogue #000664-JAX). All mice were housed together in numerical birth order in groups of mixed genotypes (three-to-five mice per cage). All the animals were housed with access to food and water *ad libitum* in a colony room kept at 19–22°C and 40%–60% humidity, under an inverted 12:12 h light/dark cycle (from 08:00 to 20:00). All animal procedures were approved by local committees [Universitat de Barcelona, CEEA (10,141) and Generalitat de Catalunya (DAAM 315/18)], in accordance with the European Communities Council Directive (86/609/EU).

### 2.2 Marine Molecules

Marine compounds were obtained from the available sample collections at the University of Barcelona. Briefly, the marine organisms were extracted with organic solvents and the extracts were further purified through chromatographic methods (HPLC) as previously reported (Núñez-Pons et al., 2010; Núñez-Pons et al., 2012). meridianins were tested as mixtures in the experiments.

### 2.3 Primary Hippocampal Neuron Cultures, Viral Transduction Fixation, and Immunofluorescence

Primary hippocampal cultures were performed as previously described (Anglada-Huguet et al., 2016). Hippocampus from E17.5 WT mouse embryos (C57BL6/J mice, MMRRC catalog #000664-JAX) were dissected and gently dissociated with a fire-polished Pasteur pipette. Cells were seeded (50,000 cells/cm^2^ for immunochemical staining) onto 24 mm culture plates pre-coated with 0.1 mg/ml poly-d-lysine (Sigma Chemical Co., St. Louis, MO, United States) and cultured in neurobasal medium supplemented with B27 (Gibco, Paisley, United Kingdom, ×50) and GlutaMAX (Gibco, ×100) at 37°C in a humidified atmosphere containing 5% CO_2_. At day *in vitro* 3, cultures were transduced with, AAV9·CamKIIα-(0.4). eGFPWPRE.rBG diluted 5,000 times (from Perelman School of Medicine, University of Pennsylvania, AV-9-PV1917). At day *in vitro* 20, the cultures were treated for 24 h with meridianins 500 nM or vehicle as previously described *in vitro* (Llorach-Pares et al., 2020). Fixed primary cultures were subjected to immunofluorescence as previously described (Giralt et al., 2017), enhancing GFP labeling using GFP FITC-conjugated antibody (1:500, #Ab6662, Abcam).

### 2.4 Tissue Fixation and Immunofluorescence

Animals were euthanized by cervical dislocation. Left hemispheres were removed and fixed for 5 days in 4% paraformaldehyde in phosphate-buffered saline (PBS). Free-floating coronal brain sections (40 μm) were obtained using a Leica vibratome (Leica VT1000S). Immunofluorescence procedure was carried out as described elsewhere (Giralt et al., 2017). The following primary antibodies were used: anti-GFAP rabbit polyclonal (1:500, Dako, REF#Z0334, Lot#20049468), anti-Iba1 rabbit polyclonal (1:500, Wako, #09-19741), and anti-APP (1:800, Novus Biologicals, #NBP2-62566). The following secondary antibody was used: Anti-rabbit AlexaFluor555 (1:300, Cat#SC-2048, Lot#L1815) from Thermo Fisher Scientific.

### 2.5 Confocal and Epifluorescence Imaging

Dorsal hippocampus in fixed tissue and fixed primary cultures were imaged using a Leica Microsystems Confocal SP5-II at the Medical School Imaging facility, with a ×20 or ×40 numerical aperture lens with 5× digital zoom and standard (one airy disc) pinhole (1 AU) when required and frame averaging (three frames per z step) were held constant throughout the study. Confocal z stacks were taken every 0.2 µm for *in vitro* experiments and every 2 µm for *in vivo* experiments, and at 1,024 × 1,024 pixel resolution. The imaging analysis was performed with the Fiji freeware (Schindelin et al., 2012). Briefly, for *in vivo* imaging analysis, for each mouse, at least two slices of 40 µm containing dorsal hippocampal tissue were analyzed. Up to two representative images, were obtained from each mouse. To estimate the density of dendritic spines in primary cultures, 31–39 dendrites from pyramidal-shaped neurons (one or two dendrites per neuron) from three different cultures were counted.

### 2.6 Immunoblot Analysis

Hippocampal samples were collected in cold lysis buffer containing 50 mM Tris base (pH 7.5), 10 mM EDTA, 1% Triton X-100, and supplemented with 1 mM sodium orthovanadate, 1 mM phenylmethylsulfonyl fluoride, 1 mg/ml leupeptin, and 1 mg/ml aprotinin. Samples were centrifuged at 16,000 g for 15 min and the supernatants collected. After incubation (1 h) in blocking buffer containing 2.5% BSA and non-fat powdered milk in Tris buffered saline Tween (TBS-T) (50 mM Tris–HCl, 150 mM NaCl, pH 7.4, 0.05% Tween 20), membranes were blotted overnight at 4°C with primary antibodies. Antibodies used for immunoblot analysis were: GSK3β (1:1,000; Cell Signaling, #9315), phosphor-GSK3β at Ser9 (1:1,000; Cell Signaling, #9336xz), BDNF (1:1,000; Icosagene, #327-100, clone 3C11), catalytic PKAα (1:1,000; Santa Cruz #sc-903), phospho-PKA at Thr197 (1:1,000 Cell Signalling #5661S), TNFα (1:500, Abcam #ab1793). The membranes were then rinsed three times with TBS-T and incubated with horseradish peroxidase-conjugated secondary antibody for 1 h at room temperature. After washing for 30 min with TBS-T, the membranes were developed using the enhanced chemiluminescence (ECL) kit (Santa Cruz Biotechnology). The ImageLab densitometry program (ImageLab from ChemiDoc system from Bio-Rad) was used to quantify the different immunoreactive bands relative to the intensity of the α-tubulin or, in the case of phospho-GSK3β and phospho-PKA, they were relativized with respect to total GSK3β and total catalytic PKAα, respectively.

### 2.7 Stereotaxic Surgery

C57BL6/J or 5xFAD mice were deeply anesthetized with ketamine-xylacine (100 and 10 mg/kg, respectively) and placed in a stereotaxic apparatus for unilateral osmotic minipump (model 1,002; Alzet, Palo Alto, CA, United States) implantation. The brain infusion kit (#0008663) was also used to deliver into the third ventricle 0.11 μl per hour of vehicle or meridianins 500 nM (0.1 mm posterior to bregma, ± 0.8 mm lateral to midline and −2.5 mm ventral to the parenchyma surface). Cannulas were fixed on the skull with the Loctite 454 (from Alzet). Minipumps, previously equilibrated overnight at 37°C in PBS, were implanted subcutaneously in the back of the animal. Mice were allowed to recover for 7 days before starting the behavioral assessment.

### 2.8 Behavioral Assessment

#### 2.8.1 Novel Object Recognition Test

The device consisted in a white square arena with 40 cm × 40 cm × 40 cm (long × wide × high, respectively). The light intensity was 40 lux throughout the arena, and the room temperature was kept at 19°C–228°C and 40%–60% humidity. Mice were first habituated to the arena in the absence of objects (1 day, 30 min). On the second day, two similar objects were presented to each mouse during 10 min (A′A″ condition), after which they were returned to their home cage. Twenty-four hours later, the same animals were retested for 5 min in the arena with a familiar and a new object (A′B condition). The object preference was measured as the time exploring each object × 100/time exploring both objects. The arena was rigorously cleaned between animal trials.

#### 2.8.2 Spontaneous Alternation in a T-Maze

The T-maze apparatus used for the T-SAT was a wooden maze consisting of three arms, two of them situated at 180° from each other, and the third, representing the stem arm of the T, situated at 90° with respect to the other two. All arms were 45 cm long, 8 cm wide, and enclosed by a 20 cm wall. The maze was thoroughly painted with waterproof gray paint. Light intensity was 5 lux throughout the maze. A 10 cm start area was situated at the end of the stem arm and closed by a wooden guillotine door. Two identical guillotine doors were placed in the entry of the arms situated at 180°. The maze was elevated 60 cm above the floor. In the training trial, one arm was closed (novel arm) and mice were placed in the stem arm of the T (home arm) and allowed to explore this arm and the other available arm (familiar arm) for 10 min, after which they were returned to the home cage. After an intertrial interval of 1 h, mice were placed in the stem arm of the T-maze and allowed to freely explore all three arms. The first choice to turn either to the familiar arm or to the new arm (alternation rate, %) was the parameter evaluated in the testing phase.

### 2.9 Golgi Staining and Dendritic Spines Counting

Fresh brain hemispheres were processed, following the Golgi–Cox method as described previously (Giralt et al., 2017). Essentially, mouse brain hemispheres were incubated in the dark for 21 days in filtered dye solution (10 g L^−1^ K_2_Cr_2_O_7_, 10 g L^−1^ HgCl2, and 8 g L^−1^ K_2_CrO_4_). The tissue was then washed 3 × 2 min in water and 30 min in 90% ethanol (EtOH) (v/v); 200 µm sections were cut in 70% EtOH on a vibratome (Leica Microsystems) and washed in water for 5 min. Next, they were reduced in 16% (v/v) ammonia solution for 1 h before washing in water for 2 min and fixation in 10 g L^−1^ Na_2_S_2_O_3_ for 7 min. After a 2 min final wash in water, sections were mounted on superfrost coverslips, dehydrated for 3 min in 50%, then 70%, 80%, and 100% EtOH, incubated for 2 × 5 min in a 2:1 isopropanol:EtOH mixture, followed by 1 × 5 min in pure isopropanol and 2 × 5 min in xylol. Brightfield images of Golgi-impregnated stratum radiatum secondary apical dendrites from hippocampal CA1 pyramidal neurons and secondary apical dendrites from cortical pyramidal neurons of the layer V in frontal cortex and entorhinal cortex were obtained. Images were captured with a Nikon DXM 1200F digital camera attached to a Nikon Eclipse E600 light microscope (×100 oil objective). Only fully impregnated pyramidal neurons with their Soma found entirely within the thickness of the section were used. Image z stacks were taken every 0.2 mm and at 1,024 × 1,024 pixel resolution, yielding an image with pixel dimensions of 49.25 × 49.25 mm. The total number of spines counting were performed by using the Fiji freeware (Schindelin et al., 2012). At least 60 dendrites per group from at least five mice per genotype were counted. Picture acquisition and subsequent analysis were performed independently by two investigators blind to genotypes, and results were then pooled. Overall differences between the results were minor.

### 2.10 Statistics

Sample sizes were determined by using the power analysis method: 0.05 alpha value, 1 estimated sigma value, and 75% of power detection. All data are expressed as mean ± SEM. Normal distribution was tested with d’Agostino and Pearson omnibus normality test. If the test was passed, statistical analysis was performed using parametric statistical analysis. Before pairs of comparisons, we performed the F test to compare variances. In experiments with normal distribution, statistical analyses were performed using the unpaired two-sided Student’s t-test (95% confidence) and the two-way ANOVA with the Bonferroni’s or Tukey’s *post-hoc* tests as appropriate and indicated in the figure legends. The t-test with Welch’s correction was applied when variances were unequal. Values of *p* < 0.05 were considered statistically significant. Grubbs and ROUT tests were performed to determine the significant outlier values. All experiments in this study were blinded and randomized by blocks of animals. All mice bred for the experiments were used for pre-planned experiments and randomized to experimental groups. Data were collected, processed, and analyzed randomly. The experimental design and handling of mice were identical across experiments. Littermates were used as controls with multiple litters (3–5) examined per experiment.

## 3 Results

### 3.1 Meridianins Increase Spine Density in Primary Cultured Hippocampal Neurons

**FIGURE 1:**
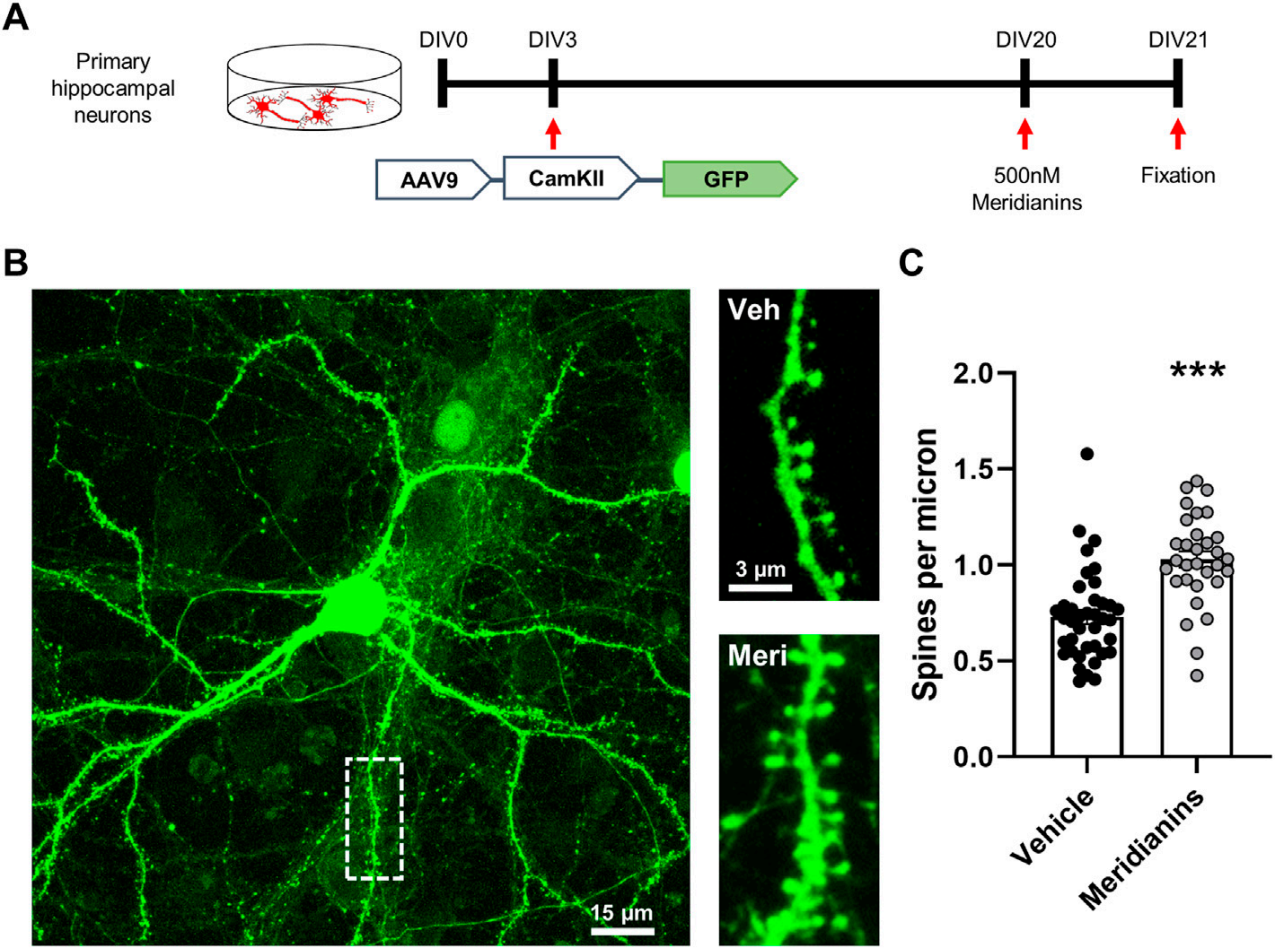
Effects of meridianins on spine density in primary hippocampal cultures. **(A)** Schematic representation of the experimental design. **(B)** Representative images showing the GFP-positive primary hippocampal pyramidal neuron (in green) at left and representative dendrites at right for both conditions, neurons treated with vehicle or with 500 nM meridianins. **(C)** Quantification of spine density is shown (Student’s *t*-test, t = 5.261, df = 68; *p* < 0.001). Data are mean ± SEM. In C, *n* = 31 and 39 dendrites/group (2 dendrites per neuron) from three different experiments. ***p < 0.001 as compared to vehicle-treated neurons.

We previously reported that meridianins are capable to induce an increase on cell arborization (Llorach-Pares et al., 2020). Thereby, we hypothesized that these compounds have the potential to induce structural synaptic plasticity. We then transduced primary hippocampal neurons with an AAV9-CaMKII-GFP at day *in vitro* 3. At day *in vitro* 21, spine density was quantified just after a 24 h treatment with meridianins 500 nM. We observed that the number of spines in meridianin-treated neurons was increased compared with neurons treated only with vehicle (Figures 1A,B). These results reveal a significant potential to induce structural synaptic plasticity by meridianins and provide a rationale for the use of these compounds to counteract cognitive decline and progressive neuropathology in AD mouse models*.*

### 3.2 Meridianins Inhibit GSK3β Activity *In Vivo*

**FIGURE 2:**
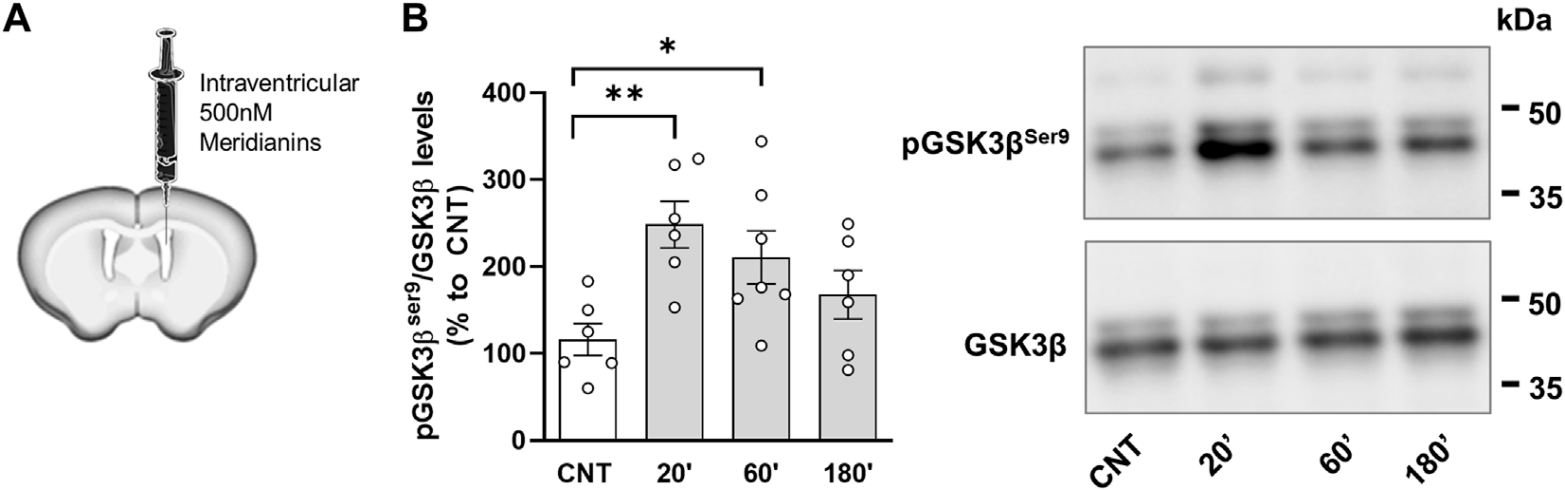
*In vivo* inhibition of hippocampal GSK3β activity. **(A)** Schematic representation of the experimental design. Nine-month-old C57BL6/J male mice were stereotaxically injected into the third ventricle with 3 μl of 500 nM meridianins. **(B)** left panel Densitometry quantification of phospho-GSK3βSer9 levels was performed in hippocampal samples upon intraventricular injection of vehicle (CNT) or at 20′ or 60′ or 180′ after intraventricular injection of Me-ridianins 500 nM. One-way ANOVA: F (3,21) = 4.373, *p* < 0.05. Data are mean ± SEM. Dunnett’s test as a *post-hoc* analysis was used. *p < 0.05; **p < 0.01; compared with CNT mice. **(B)** right panel Representative immunoblotting for phospho-GSK3βSer9 and total GSK3β as a loading control. Molecular weight markers’ positions are indicated in kDa in B (*n* = 6–7/group).

Although we already reported that meridianins can inhibit GSK3β *in vitro* (Llorach-Pares et al., 2020), this potential function has never been tested *in vivo*. From the same previous report, *in silico* simulations suggested that meridianins could have difficulties to cross the blood–brain barrier. Thus, we first verified whether meridianins could exert GSK3β inhibition *in vivo* by injecting the compound in the third ventricle of the brain in living adult mice (Figure 2A). We evaluated GSK3β inhibition in the hippocampal tissue by estimating the phosphorylation levels of the protein in the serine 9 residue (Ser9), which is widely established to be a substrate directly related with GSK3β inhibition (Fang et al., 2000). We observed that GSK3β inhibition in the hippocampus was significant 20 and 60 min after intraventricular injection of meridianins 500 nM (Figure 2B), as observed by an increase in the phosphorylation levels at the Ser9 residue of the protein. These results encouraged us to evaluate whether meridianins could improve or ameliorate cognitive deficits in the 5xFAD mouse model of AD.

### 3.3 Meridianins Improve Object Recognition Memory and Cognitive Flexibility in the 5xFAD Mouse Model

**FIGURE 3:**
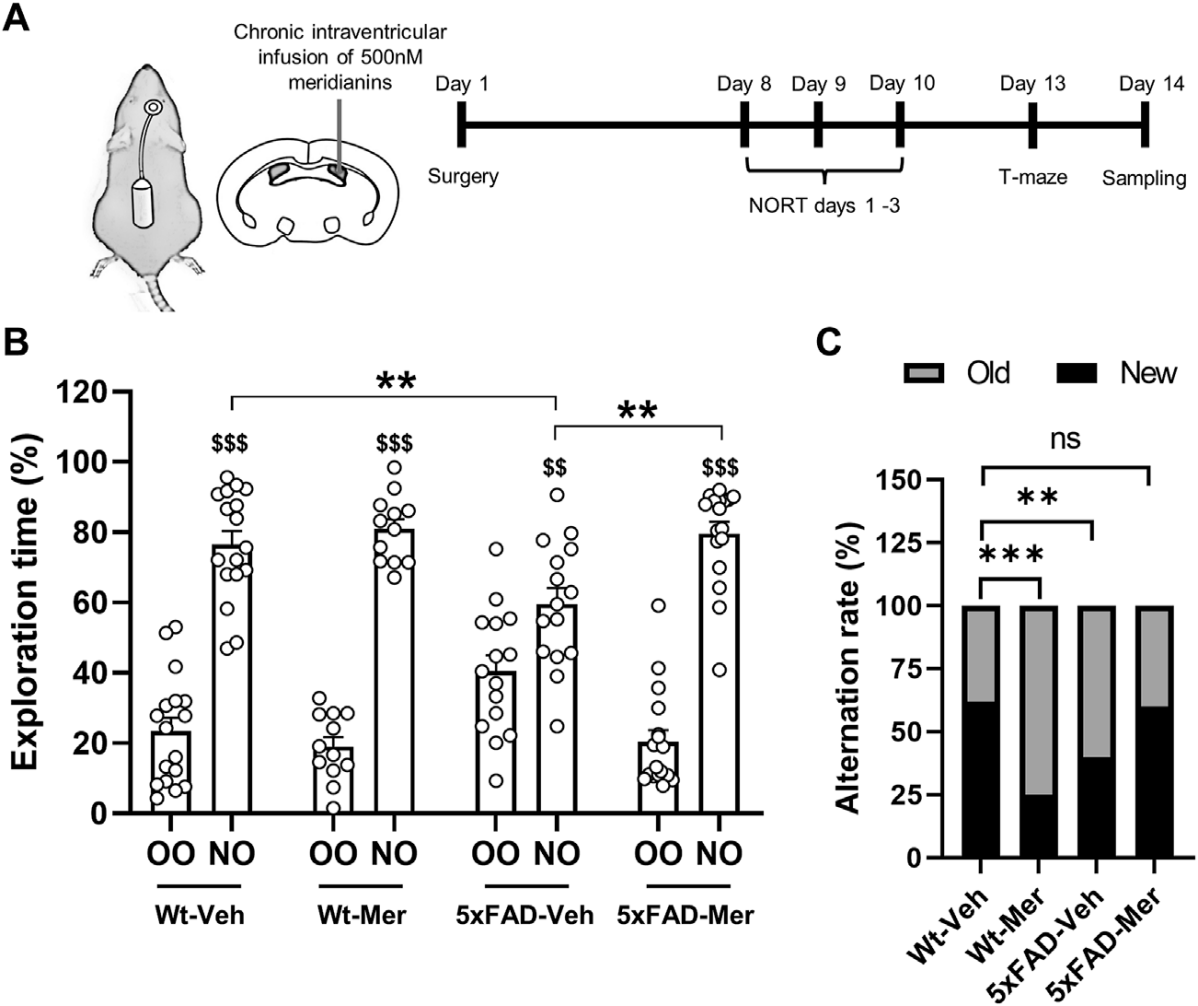
Cognitive effects of meridianins’ chronic delivery in the hippocampus of 9-month-old 5xFAD mice. **(A)** Schematic representation of the experimental design. Nine-months-old WT and 5xFAD mice were chronically treated for 14 days with 500 nM meridianins delivered into the third ventricle. **(B)** The same WT and 5xFAD mice were subjected to the NORT task. Preference for an original object (OO, old object) and a new object (NO, new object) was quantified 24 h after training. Graphs show the percentage of object preference in WT and 5xFAD mice during object recognition memory assessment (Preference effect: F (1,114) = 321.1, *p* < 0.001; Interaction effect: F (3,114) = 13.31, *p* < 0.001). Data are mean ± SEM. Tukey’s test as a *post-hoc* analysis was used. **p < 0.01 compared with novel object preference percentage in 5xFAD vehicle mice; $$ *p* < 0.01 and $$$ *p* < 0.001 comparing novel object preference with old object preference in each group. **(C)** Cognitive flexibility was measured by the spontaneous alternation learning assessed by the T-SAT in the same 9-month-old WT and 5xFAD mice. Animals were tested for spontaneous alternation rate, evaluating the percentage of correct choices in the testing phase of the T-SAT (chi-square, df: WT Veh vs. WT Meri, 27.85, 1, *p* < 0.001; WT Veh vs. 5xFAD Veh, 9.684, 1, *p* < 0.01; WT Veh vs. 5xFAD Meri = 0.084, 1, *p* = 0.771). Number of animals per group: WT Veh: 17, WT Mer: 12, 5xFAD Veh: 15, 5xFAD Mer: 17.

The 5xFAD transgenic mice display a strong phenotype with a relatively fast time course compared with other AD mouse models. We have previously observed that 8-month-old 5xFAD mice already display deficits in novel object recognition memory and in cognitive flexibility measured by the NORT and the spontaneous alternation in a T-maze or T-SAT (Giralt et al., 2018; de Pins et al., 2019; Pérez-Sisqués et al., 2021). Thus, we implanted in 9-month-old WT and 5xFAD mice a mini-osmotic pump to continuously deliver vehicle or meridianins 500 nM into the third ventricle of the brain for 14 days (Figure 3A). Seven days after surgical intervention, WT and 5xFAD mice treated with vehicle or meridianins were subjected first to the NORT and then to the T-SAT. First, in the NORT, we observed that both WT and 5xFAD mice explored more the new object with respect to the old one (Figure 3B). However, the preference of 5xFAD mice treated with vehicle for the new object was significantly reduced compared with that registered in 5xFAD mice treated with meridianins. These results indicate the presence of cognitive deficits in 5xFAD treated with vehicle compared with WT treated with vehicle but that such deficits were rescued in 5xFAD mice treated with meridianins (Figure 3B). In the T-SAT task, the spontaneous alternation was evaluated 1 h after a training session. WT vehicle and 5xFAD meridianins mice significantly alternated, whereas 5xFAD vehicle mice did not (Figure 3C). Intriguingly, WT meridianins also showed impaired spontaneous alternation, suggesting that permanent GSK3β inhibition in healthy animals can be deleterious for some cognitive skills. In summary, meridianins rescued the observed impairments on object recognition memory and spontaneous alternation in the 5xFAD mice.

### 3.4 Meridianins Rescue Synaptic Loss in the Entorhinal Cortex and in the Hippocampus of the 5xFAD Mouse Model

**FIGURE 4:**
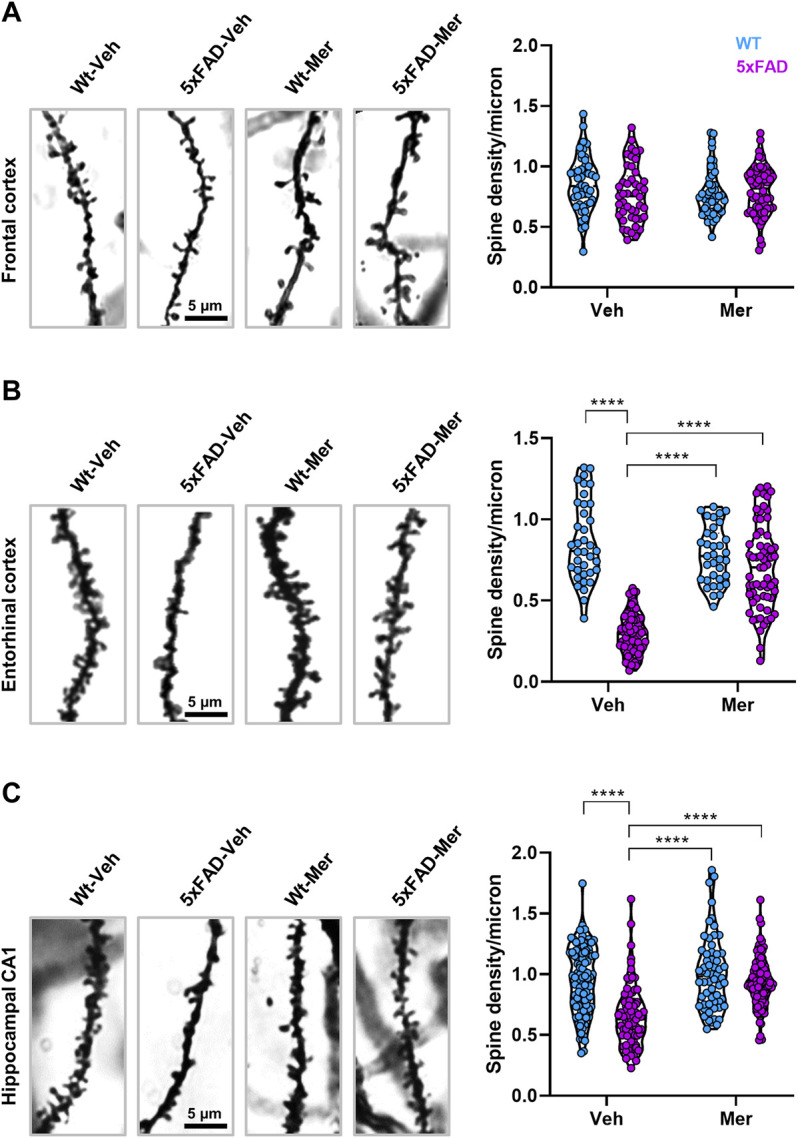
Dendritic spine density in the frontal cortex, entorhinal cortex, and hippocampus of WT and 5xFAD treated with vehicle or meridianins. Postmortem brain samples from the mice used in the experiment conducted in Figure 3 were used for Golgi staining. **(A)** Images of second-order apical dendrites from pyramidal neurons of the layer V in frontal cortex stained with Golgi (Left panel) staining in 9-month-old WT and 5xFAD treated with vehicle or meridianins 500 nM. The dendritic spine density (right panel) was determined in all four groups. Two-way ANOVA found no differences between groups or treatments. **(B)** Images of second-order apical dendrites from pyramidal neurons of the layer V in entorhinal cortex stained with Golgi (left panel) staining in 9-month-old WT and 5xFAD treated with vehicle or meridianins 500 nM. The dendritic spine density (right panel) was determined in all four groups. Two-way ANOVA (group effect: F (1,204) = 114.1, *p* < 0.001; interaction effect: F (1,204) = 67.06, *p* < 0.001). **(C)** Images of second-order apical dendrites from pyramidal neurons of the hippocampal CA1 stained with Golgi (left panel) staining in 9-month-old WT and 5xFAD treated with vehicle or meridianins 500 nM. The dendritic spine density (right panel) was determined in all four groups. Two-way ANOVA [group effect: F (1,336) = 43.3, *p* < 0.001; interaction effect: F (1,336) = 14.55, *p* < 0.001]. Data are mean ± SEM. Tukey’s test as a *post-hoc* analysis was used. *p < 0.05, **p < 0.01 and ***p < 0.001. Number of samples in **(A)**: 45–62 dendrites/group (from 5 mice/group); **(B)**: 35–72 dendrites/group (from 5 mice/group); and **(C)**: 72–107 dendrites/group (from 5 mice/group).

Since we observed a significant enhancement of synaptic density in cultured hippocampal neurons upon meridianin treatment, we then hypothesized that the potential cognitive improvements displayed by 5xFAD mice treated with meridianins could be associated to an induction of structural synaptic plasticity. We then performed a Golgi staining in all four groups of mice, and spine density analysis was carried out in the frontal cortex, entorhinal cortex, and CA1 of the hippocampus. We analyzed three regions because meridianins were delivered into the third ventricle and, consequently, many brain regions could be reached by the compound. First, we did not observe any change from any group with regard to the spine density in layer V pyramidal neurons of the frontal cortex (Figure 4A). In contrast, spine density was decreased in layer V pyramidal neurons of the entorhinal cortex in 5xFAD mice treated with vehicle whereas spine density was completely rescued in 5xFAD mice treated with meridianins (Figure 4B). Similarly, spine density was also decreased in CA1 pyramidal neurons of the hippocampus in 5xFAD mice treated with vehicle whereas spine density was completely rescued in 5xFAD mice treated with meridianins (Figure 4C). Taken altogether, meridianins rescued synapse loss in 9-month-old 5xFAD mice in, at least, two brain regions, namely, entorhinal cortex and hippocampal CA1.

### 3.5 Meridianins Ameliorate Microgliosis and Partially Astrogliosis but do not Exert Effects in Plaque Number in the 5xFAD Mouse Model

**FIGURE 5:**
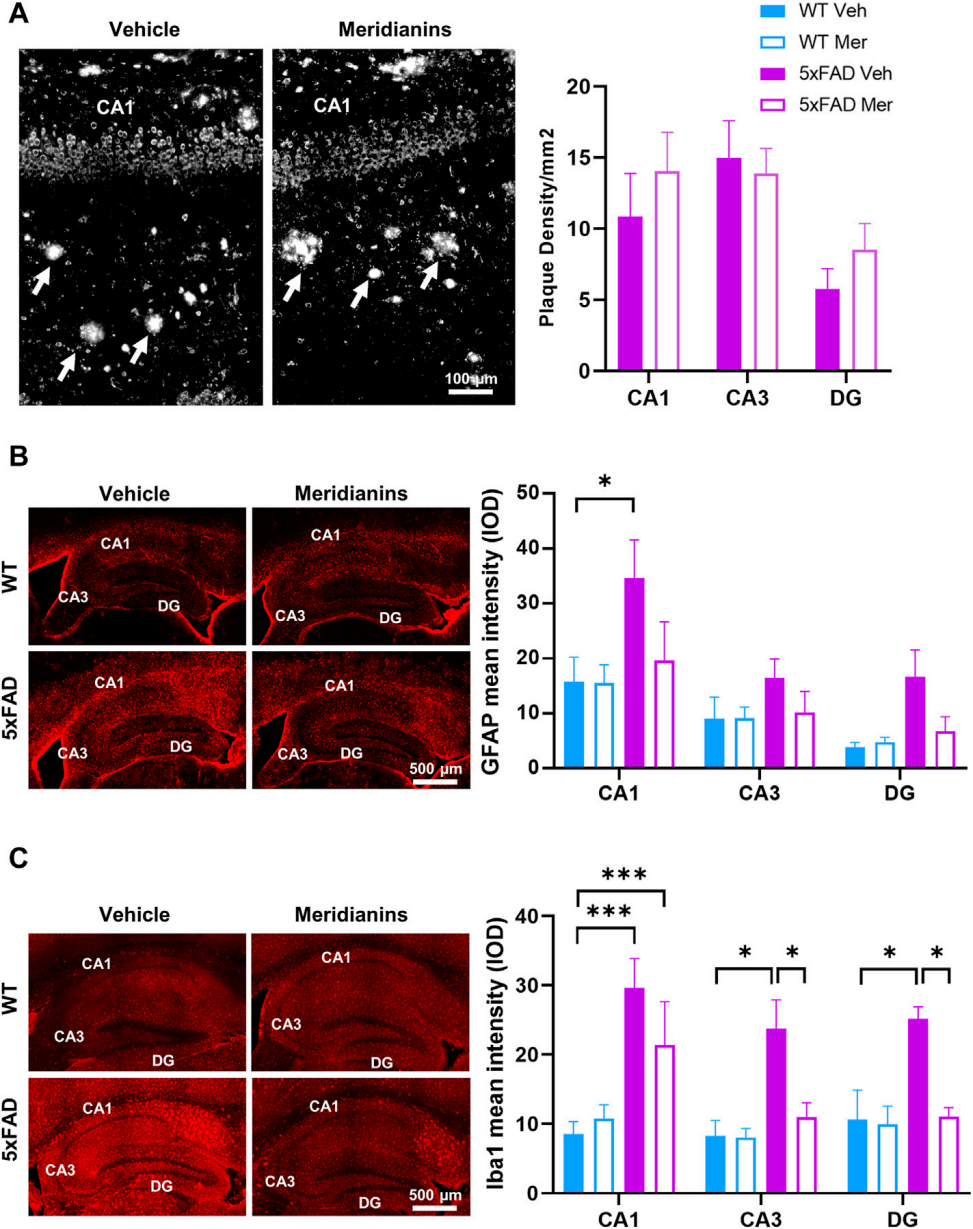
Plaque numbers and GFAP and Iba1 levels in the hippocampus of WT and 5xFAD treated with vehicle or meridianins. Postmortem brain samples from the mice used in the experiment conducted in panel 3 were used for immunofluorescences against Iba1, GFAP, and amyloid plaques. **(A)** Amyloid plaques images stained obtained in a bright-field microscope in the hippocampus (left panel) of 5xFAD treated with vehicle or meridianins 500 nM. Representative images from CA1 are depicted. White arrows point at representative plaques. The plaque density was determined by manual counting in the CA1, CA3, and DG in the hippocampus of 5xFAd treated with vehicle or with meridianins (right panel). Paired comparisons using Student’s *t*-test did not detect any statistical difference between the two groups in any hippocampal subarea. **(B)** GFAP immunofluorescence microscopy imaging in the dorsal hippocampus of 9-month-old WT and 5xFAD mice treated with vehicle or meridianins (left panel). Quantification of GFAP relative intensity in the CA1, CA3, and DG in the four groups (right panel, two-way ANOVA, group effect: F F (2,84) = 11.47, *p* < 0.001). **(C)** Iba1 immunofluorescence microscopy imaging in the dorsal hippocampus of 9-month-old WT and 5xFAD mice treated with vehicle or meridianins (left panel). Quantification of GFAP relative intensity in the CA1, CA3, and DG in the four groups [right panel, two-way ANOVA, group effect: F (3,76) = 14.48, *p* < 0.001]. Data are mean ± SEM. Tukey’s test as a *post-hoc* analysis was used. *p < 0.05 and ***p < 0.001. Number of samples in **(A)**: 6–7; in **(B)**: 6–9 and in **(C)**: 6–9. CA1–3: cornu ammonis 1–3, DG: dentate gyrus.

Since we observed a prominent improvement on the synapse loss and recovery of some cognitive functions, we then sought for potential improvements in hippocampal neuropathological hallmarks such as the plaque number, astrogliosis and microgliosis. First, the plaque number did not differ between 5xFAD mice treated with vehicle and 5xFAD mice treated with meridianins in any hippocampal subregion, namely, CA1, CA3, or DG (Figure 5A). Next, we evaluated astrogliosis in all four groups by evaluating GFAP staining in all three hippocampal subregions: CA1, CA3, and DG (Figure 5B). Our analysis showed a trend of increased GFAP in all three subareas in 5xFAD mice treated with vehicle. However, this increase was significant only in the CA1 when compared with WT treated with vehicle. Interestingly, 5xFAD mice treated with meridianins did not show changes in GFAP staining in any hippocampal subarea compared to WT mice treated with vehicle. We next measured Iba1 staining intensity as a marker of microgliosis (Figure 5C) in all four groups. Changes in microgliosis using Iba1 as a marker were more prominent and clearer. Concretely, the intensity of Iba1 staining was upregulated in the 5xFAD mice treated with vehicle in all three hippocampal subareas (CA1-DG). In contrast, the intensity of Iba1 staining was upregulated only in the CA1 but not in CA3 and DG in 5xFAD mice treated with meridianins. Altogether, these results suggest that meridianins do not affect the plaque number, but it ameliorates neuroinflammation in the hippocampus of 5xFAD mice.

### 3.6 Biochemical Effects Mediated by Meridianins in the 5xFAD Mouse Model

**FIGURE 6:**
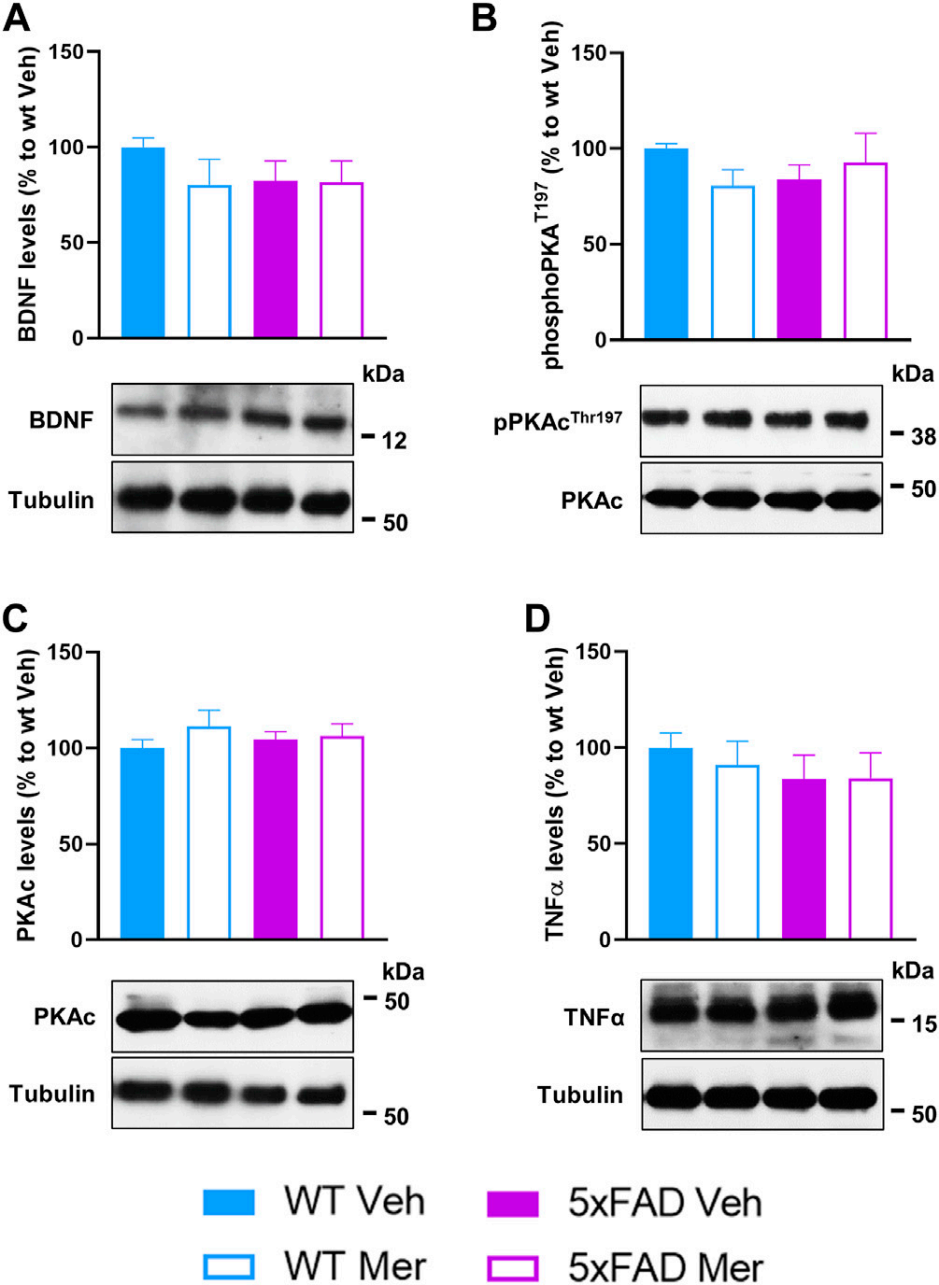
Biochemical assessment of 5xFAD mice treated with meridianins. **(A)** Densiometry quantification of BDNF levels with tubulin as a loading control. **(B)** Densitometry quantification of phospho-PKAthr197 levels with total catalytic PKA as a loading control. **(C)** Densitometry quantification of total catalytic PKA levels with tubulin as a loading control. **(D)** Densitometry quantification of TNFα levels with tubulin as a loading control. All Western blots were performed in hippocampal samples from WT and 5xFAD mice treated with vehicle or meridianins 500 nM as shown in the experimental design from panel 3. Molecular weight markers’ positions are also indicated in kDa (*n* = 10–14/group).

Since we observed a restoration of synapse loss, we then tested for a potential concomitant effect in the PKA-CREB-BDNF pathway in the hippocampus of 5xFAD mice treated with meridianins. For this purpose, we performed Western blot experiments in hippocampal lysates from all four groups of mice that underwent behavioral characterization (Figure 3) to evaluate total protein levels for BDNF (Figure 6A), phosphorylation levels of PKA in the threonine 197 (Figure 6B), and total catalytic PKA levels (Figure 6C), but no changes were observed in any group compared with WT mice treated with vehicle. To further investigate biochemical changes related with neuroinflammation induced by meridianins in the hippocampus, we interrogated TNFα levels (Figure 6D). We did not detect differences in any group compared with WT mice treated with vehicle. We concluded that meridianins may exert their effects through alternative pathways to ultimately rescue the loss of excitatory synapses and to ameliorate microgliosis and astrogliosis in 5xFAD mice.

## 4 Discussion

In a previous report, we observed that meridianins can inhibit GSK3β in primary neuronal cultures without altering the cell viability (Llorach-Pares et al., 2020). Therefore, in the present work, we hypothesized that meridianins could inhibit GSK3β *in vivo* and to exert beneficial improvements in a mouse model of AD. Thus, we first observed *in vitro* that meridianins induce structural synaptic plasticity in primary hippocampal neurons and that its intracerebral administration in living mice inhibits GSK3β in the hippocampal region. These promising results prompted us to chronically administer meridianins into the brain of the 5xFAD mouse model of AD. Interestingly, we observed that this treatment induced robust positive changes in synaptic density in two main affected brain regions in AD, namely, entorhinal cortex and hippocampus accompanied with a significant reduction of neuroinflammatory processes such as astrogliosis and microgliosis in the hippocampus of these mice. All these improvements were associated with a correction of object recognition and spontaneous alternation deficits commonly displayed by these mice (Oakley et al., 2006; Giralt et al., 2018; de Pins et al., 2019; Pérez-Sisqués et al., 2021).

Our observations indicate that meridianins are potent inhibitors of GSK3β *in vitro* (Llorach-Pares et al., 2020) and *in vivo* (present results). Such inhibition was associated with a robust increase of dendritic spines both *in vivo* and *in vitro*, which is in agreement with previous studies showing that GSK3β inhibition may induce spinogenesis (Jorge-Torres et al., 2018). The latter phenomenon could probably be modulated by changes in molecular processes like actin polymerization dynamics in dendritic spines (Cymerman et al., 2015), which in turn could alter the presence and function of essential receptors involved in synaptic plasticity such as α-amino-3-hydroxy-5-methyl-4-isoxazolepropionic acid receptors in the plasma membrane of these microstructures (Ochs et al., 2015). Furthermore, it is noteworthy that excessive GSK3β inhibition/deletion can induce spine loss (Liu et al., 2017) as well as its overexpression (Pallas-Bazarra et al., 2017). Similarly, deficient mice for GSK3β in specific neuronal subpopulations as well as its overexpression in particular neuronal types induce learning and memory impairments in mice (Jaworski et al., 2019). The later reports highlight the fact that GSK3β manipulation must be highly controlled and probably with only moderate levels of modulation. In this line, one of the most studied GSK3β inhibitors and already used in humans for the treatment of bipolar disorder, lithium, also acts indirectly by enhancing the serine phosphorylation of GSK3β (Freland and Beaulieu, 2012). Lithium treatment alleviates memory deficits in mice expressing both APP and PS1 (Zhang et al., 2011). However, unclear results have been found in clinical studies using lithium in AD patients with some of them detecting cognitive improvements (Forlenza et al., 2011), whereas others did not (Hampel et al., 2009). Interestingly, a retrospective study of bipolar- and unipolar-depression patients with a history of lithium treatment found that these patients had a higher risk of developing dementia (Dunn et al., 2005). Therefore, it appears that lithium as a treatment for AD remains unconclusive. In the present study, we found that meridianins at the dose used are in a correct balance to avoid detrimental effects caused by excessive GSK3β manipulation, reinforcing the need to further investigate its applicability as a potential treatment for AD in the near future.

Regarding the neuroinflammatory processes largely described in the 5xFAD mice and in human subjects with AD (Oakley et al., 2006; Heneka et al., 2015), it is worth mentioning that GSK3β activation has been associated with increases in neuroinflammation such as astrogliosis and microglial activation (Wang et al., 2010; Ko et al., 2014). This aberrant astroglial and microglial activation could in turn alter the synaptic connectivity by, for example, removing synapses *via* processes such as synaptic engulfment (Rajendran and Paolicelli, 2018). Thereby, it is also probable that the reduction of microglia and astroglia aberrant activities induced by meridianins would be a first step for the synaptic repair and the consequent significant cognitive restoration in 5xFAD mice.

Finally, although we demonstrated a GSK3β inhibition by meridianins, we cannot rule out the existence of alternative mechanisms of action by which meridianins could induce structural synaptic plasticity and memory improvements in the 5xFAD mice. Additionally, since AD is a multifactorial neurotoxic disease, future therapeutic strategies should not only target GSK3β but also be combined with other types of interventions targeting other core molecules and processes (Kramer et al., 2012). Overall, challenging parameters related to meridianins’ potential mechanism of action, druggability, pharmacokinetics, safety, and its capacity to inhibit GSK3β have previously been overcome (Llorach-Pares et al., 2020). Furthermore, induced structural synaptic plasticity, the reduction of neuroinflammatory processes, and rescue of memory deficits in a preclinical mouse model of AD have been successfully addressed in the present work. Finally, since meridianins do not cross the blood–brain barrier, we conclude that future studies should be carried out to generate safe and effective meridianin-based synthetic compounds to use them in preclinical and clinical studies with the final goal to be used in the treatment of AD.

## Data Availability

The raw data supporting the conclusion of this article will be made available by the authors, without undue reservation.
